# The physiology and psychology of negative splits: insights into optimal marathon pacing strategies

**DOI:** 10.3389/fphys.2025.1639816

**Published:** 2025-07-16

**Authors:** Gerasimos V. Grivas

**Affiliations:** Physical Education and Sports, Division of Humanities and Political Sciences, Hellenic Naval Academy, Piraeus, Greece

**Keywords:** negative splits, marathon pacing, endurance performance, thermoregulation, cardiovascular drift

## Abstract

Pacing strategy plays a critical role in marathon performance, influencing both physiological stress and race outcomes. Among the available pacing approaches, the negative split running the second half faster than the first has emerged as a potentially optimal strategy for endurance athletes. This mini-review explores the physiological mechanisms and psychological factors underpinning the effectiveness of negative splits in marathon running. Key advantages include improved energy conservation and glycogen sparing, enhanced thermoregulation, and reduced cardiovascular drift. These benefits contribute to delayed fatigue and greater efficiency in the latter stages of the race. Training modalities such as progressive long runs and fast-finish workouts, along with psychological techniques like goal setting and visualization, are discussed as tools to help runners implement negative split strategies. The article also examines the limitations and applicability of negative splits among elite and recreational runners, highlighting the need for individualized approaches based on fitness level, environmental conditions, and race profile. In conclusion, while not universally applicable, negative splits represent a scientifically grounded pacing method that may optimize performance in marathon running across a range of athlete populations.

## 1 Introduction

Marathon running performance is determined by a complex interplay of physiological, psychological, and environmental factors. Among various pacing strategies employed by runners, the negative split, where the second half of the race is completed faster than the first has garnered increasing attention from scientists and coaches. While positive and even pacing strategies are common, elite marathoners frequently demonstrate a slight negative split pattern, suggesting potential physiological benefits (Hanley, 2015; Cuk et al., 2021).

Recent reviews have emphasized the physiological and psychological mechanisms underpinning pacing behaviors in the marathon, reinforcing the value of well-structured pacing strategies for optimal performance (Nikolaidis and Knechtle, 2018; Nikolaidis and Knechtle, 2024). Negative splitting requires precise control of energy expenditure, thermoregulation, neuromuscular function, and psychological pacing awareness. From a physiological perspective, such a strategy may optimize muscle glycogen utilization, reduce early accumulation of metabolic byproducts, and delay the onset of fatigue. Furthermore, runners who adopt conservative early pacing may maintain better biomechanical form and cardiovascular efficiency later in the race (Abbiss and Laursen, 2008).

Despite its potential benefits, negative splitting remains underutilized among recreational runners, who often succumb to early overpacing and positive splits, compromising performance and increasing the risk of exhaustion. Understanding the physiological mechanisms that underlie negative split performance can help guide training interventions and pacing education tailored to runner experience and goal setting (Foster et al., 1994).

This mini review aims to summarize the key physiological and psychological factors that support the negative split pacing strategy in marathon running. I also explore practical implications for training and racing while addressing limitations and individual considerations for both elite and recreational athletes.

## 2 What are negative splits and why do they matter?

Pacing strategy is a critical determinant of marathon performance, influencing physiological stress, energy availability, and race outcomes. Among the primary pacing strategies positive, even, and negative splits, the negative split is characterized by a faster second half of the race compared to the first. This approach contrasts with the more common positive split pattern, in which runners start aggressively and gradually slow down due to fatigue and depleted energy reserves (Abbiss and Laursen, 2008).

The theoretical advantage of negative splits lies in their alignment with the physiology of fatigue. By starting conservatively, runners reduce the risk of early glycogen depletion and excessive accumulation of lactate and hydrogen ions, preserving muscular efficiency and delaying central fatigue (Foster et al., 1994). Moreover, this strategy minimizes cardiovascular drift and allows for better thermoregulatory control during the hotter and more fatiguing final stages of the race (Nybo et al., 2014).

Empirical data support the effectiveness of negative splits in elite performance. Analyses of world-class marathon events show that a majority of record-breaking performances follow either an even or slight negative split profile (Hanley, 2015). For instance, Eliud Kipchoge’s sub-2-h marathon attempt involved a highly controlled pace with subtle acceleration in the final stages, a textbook example of even-to-negative pacing (Joyner et al., 2011).

However, despite its documented benefits, negative splitting requires discipline, experience, and precise internal regulation qualities often lacking in less experienced runners. As a result, recreational athletes tend to exhibit positive splits, which correlate with poorer performance and higher perceived exertion in the final segments of the race (Buman et al., 2008).

Understanding the physiological rationale behind negative splits provides a foundation for optimizing training and race-day strategy, especially in endurance sports where energy management is paramount.

## 3 Physiological mechanisms supporting negative splits

### 3.1 Energy conservation and glycogen sparing

One of the most critical determinants of marathon performance is the availability of muscle glycogen. Starting a race too fast increases the rate of glycogen breakdown through anaerobic glycolysis, leading to premature depletion and the accumulation of fatigue-inducing metabolites such as lactate and hydrogen ions (Coyle, 1995). A negative split pacing strategy, by promoting conservative early pacing, allows for more gradual glycogen utilization, thus preserving energy reserves for the final stages of the race (Sha et al., 2024). This approach has been consistently associated with superior race outcomes, as it helps runners maintain a more even pace and avoid the sharp decline in performance often seen in positive splits. Recent studies have demonstrated that athletes who adopt a negative split strategy tend to achieve better overall times and experience less physiological disruption in the latter stages of the race (Pycke and Billat, 2022; Sha et al., 2024). Moreover, pacing variations in the early stages have been strongly linked to performance outcomes in both elite and recreational marathon runners (Ristanović et al., 2023).

Slower initial running speeds are associated with a higher proportion of energy being derived from fat oxidation, sparing glycogen stores for when they are most needed. This is particularly important because once glycogen is depleted, runners are forced to rely on slower, less efficient energy systems often referred to as “hitting the wall” or experiencing hypoglycemia-induced fatigue (Bosquet et al., 2002). By delaying this point of metabolic crisis, runners employing a negative split are more likely to maintain velocity and avoid the sharp performance decline observed in positive split patterns.

Moreover, glycogen preservation may help sustain neuromuscular coordination and delay both central and peripheral fatigue, which are essential for maintaining biomechanical efficiency in the later stages of a marathon (Hargreaves and Spriet, 2020). In well-trained runners, prolonged exercise (90–120 min) has been shown to significantly impair running economy and VO_2peak_, indicative of neuromuscular and metabolic limitations (Zanini et al., 2025). As fatigue accumulates, runners often experience deteriorations in movement patterns, including increased ground contact time, altered stride mechanics, and suboptimal muscle activation. These changes can impair running economy and elevate the risk of injury (Darch et al., 2022). Recent field-based studies in adult distance runners have confirmed such fatigue-induced biomechanical alterations. For example, Prigent et al. (2022) observed that recreational runners during a half-marathon exhibited progressive increases in ground contact time and reductions in stride frequency as fatigue developed. Similarly, a meta-analysis by Hazzaa et al. (2023) highlighted consistent kinematic and plantar pressure changes associated with muscle fatigue, reinforcing the link between energy depletion and compromised running mechanics. By delaying the onset of these fatigue-related adaptations, conservative pacing strategies may help preserve efficient form, reduce unnecessary energy expenditure, and lower injury risk in the final stages of the race.

Overall, from a bioenergetic standpoint, negative splits align with the physiological principle of pacing energy output in accordance with substrate availability and muscular endurance capacity, which is an especially critical concept in prolonged endurance events such as the marathon (Table 1).

**TABLE 1:** Physiological effects of conservative start on energy metabolism.

Physiological mechanism	Impact on performance
Reduced glycolytic flux at race start	Slower glycogen depletion, better energy control
Greater reliance on fat oxidation	Energy preservation for late race
Delayed accumulation of lactate and H+ ions	Reduced early fatigue
Prolonged glycogen availability	Improved endurance in later stages
Preserved neuromuscular efficiency	Maintained running form and reduced injury risk

### 3.2 Thermoregulation and fatigue onset

Thermoregulation plays a vital role in endurance performance, particularly in the marathon where prolonged effort leads to progressive heat accumulation. As core body temperature rises, the central nervous system initiates protective mechanisms to limit further exertion, a phenomenon often referred to as “central fatigue” (Nybo and Nielsen, 2001). A negative split strategy, characterized by lower initial intensity, allows for a more controlled rate of heat production, helping to delay the onset of thermally induced fatigue.

Starting at a conservative pace reduces metabolic heat generation during the early stages of the race, minimizing the strain on heat dissipation mechanisms such as cutaneous vasodilation and sweating. This is especially relevant in warm or humid environments, where the body’s ability to offload heat is compromised, and the risk of hyperthermia increases (González-Alonso et al., 1999). By avoiding an early surge in core temperature, runners can maintain better thermal balance and avoid the performance decline often observed when core temperature exceeds critical thresholds (typically around 40°C) (Nybo, 2008). Recent data from the Brighton Marathon confirmed this concern, as recreational runners exhibited a progressive rise in core body temperature throughout the race, with several exceeding 39.5°C in the final stages underscoring the need for pacing strategies that delay thermoregulatory fatigue (Grivas et al., 2024).

Moreover, better thermoregulation contributes to improved cardiovascular stability. In hot conditions, cardiac output is partly redirected toward the skin for cooling purposes, which can compromise muscle perfusion if intensity is too high early on. A negative split strategy minimizes this competition by keeping heart rate and internal temperature more stable, allowing for more efficient oxygen delivery to working muscles in the later stages of the race.

Thus, from a thermophysiological perspective, negative splits not only preserve energy but also help maintain homeostasis, delaying both central and peripheral fatigue under demanding environmental conditions (Table 2).

**TABLE 2:** Thermoregulatory considerations in negative split marathon pacing.

Thermoregulatory factor	Implication for negative splits
Core temperature rise	Controlled early pace reduces rate of heat accumulation
Central fatigue	Delayed due to lower thermal stress
Cutaneous vasodilation	Less demand early in race improves later cooling capacity
Sweating mechanism	Preserved function reduces risk of dehydration
Environmental stress (heat/humidity)	Negative splits mitigate hyperthermia risk
Cardiovascular competition (skin vs. muscle)	Maintains perfusion to muscles by reducing early thermal load

In addition to thermoregulatory and metabolic factors, exercise-induced muscle damage has also been proposed as a contributing factor to the decline in running pace during the latter stages of a marathon. Del Coso et al. (2013) reported that runners with elevated markers of muscle damage (e.g., creatine kinase levels) exhibited greater pacing variability and a more pronounced reduction in speed. These findings suggest that structural disruption of muscle fibers may exacerbate fatigue and impair biomechanical efficiency, particularly under conditions of depleted energy reserves and increased thermal strain. Incorporating this perspective further supports the rationale for conservative early pacing, as it may help mitigate mechanical stress and preserve muscular integrity for the final segments of the race.

### 3.3 Cardiovascular stability and pacing regulation

Cardiovascular responses to prolonged endurance exercise play a central role in sustaining performance, especially in events like the marathon. One of the key phenomena observed during prolonged running is cardiovascular drift a gradual increase in heart rate and decrease in stroke volume at a constant workload, typically driven by dehydration, rising core temperature, and prolonged sympathetic activation (Coyle and González-Alonso, 2001). A negative split pacing strategy can help attenuate this drift by minimizing early cardiac stress and preserving cardiovascular efficiency for the latter half of the race.

By starting at a more moderate pace, runners reduce early strain on the heart and delay the rise in heart rate, thereby maintaining a more stable cardiac output over time. This allows for more efficient oxygen transport to working muscles and helps avoid premature fatigue related to circulatory insufficiency. Moreover, delayed cardiovascular drift is associated with improved maintenance of VO_2_ kinetics, meaning that a runner can sustain a given pace more economically in the latter stages of the race.

Another important aspect of cardiovascular pacing is perceived exertion. Conservative early pacing leads to a lower rating of perceived exertion (RPE) in the first half, which tends to rise linearly throughout the race rather than peaking too early. This psychological pacing feedback allows the athlete to better manage effort and make more strategic decisions when increasing pace in the final kilometers (Renfree et al., 2012).

Although negative split pacing offers clear physiological and biomechanical advantages, it is not universally optimal. Recent research highlights substantial individual variability in pacing responses. Recreational runners tend to exhibit greater fluctuations and are more sensitive to pacing dynamics, whereas elite runners maintain highly consistent pacing profiles across different races (Cuk et al., 2021; Oficial-Casado et al., 2022). These findings emphasize the importance of context-specific pacing strategies that take into account the athlete’s experience, performance level, and race conditions.

Ultimately, negative splits enable more balanced cardiovascular regulation, which supports both physiological function and psychological control. In contrast, aggressive early pacing may lead to early cardiovascular strain and reduce performance reserves when they are needed most (Table 3).

**TABLE 3:** Cardiovascular stability and pacing regulation in negative split strategies.

Physiological/psychological aspect	Effect of negative split strategy
Cardiovascular drift	Attenuated due to lower early heart rate and reduced thermal and dehydration stress
Heart rate response	Delayed rise in HR allows more stable cardiac output during the race
VO_2_ kinetics	Improved maintenance of VO_2_ kinetics in later stages of race
Oxygen delivery	More efficient transport of oxygen to working muscles
Perceived exertion	Lower RPE in early stages; gradual increase supports effort regulation
Performance reserve	Preserved cardiovascular reserve for final push in race

## 4 Training and psychological strategies for achieving negative splits

Successfully executing a negative split strategy on race day requires more than just physiological readiness it demands precise pacing control, mental discipline, and targeted training that simulates the physiological demands of a progressive effort. While elite athletes may adopt negative splits instinctively, most recreational runners benefit from structured preparation and psychological conditioning.

From a training perspective, progressive long runs serve as an effective tool to simulate negative split pacing. These sessions begin at an easy pace and gradually build to race pace or faster in the final kilometers, allowing the athlete to rehearse the metabolic and mental transition from conservative to assertive effort (Foster et al., 1994; Midgley et al., 2006). Tempo runs with a fast finish, negative split intervals, and controlled progression runs are additional methods to develop both pacing control and late-race strength (Esteve-Lanao et al., 2007).

Equally important is the development of pacing awareness through regular training with heart rate monitors, GPS pacing, or perceived exertion scales. Teaching athletes to identify and internalize the sensations associated with various intensities can reduce the likelihood of early overpacing a common cause of positive splits among novice runners (Tucker et al., 2006; Renfree and St Clair Gibson, 2013).

On the psychological side, negative splitting requires confidence, patience, and a tolerance for initial underperformance. Runners must resist the temptation to “bank time” early and instead trust in their ability to finish strong. Techniques such as visualization, goal setting, mindfulness, and self-talk can help reinforce a conservative start and maintain focus during the crucial latter stages of the race (Barwood et al., 2008; Brick et al., 2015; McCormick et al., 2015).

Furthermore, race-day strategy should include course familiarization, environmental planning, and fueling strategies that align with a negative split approach. The integration of physiological training and psychological readiness is essential for translating pacing theory into successful performance (Table 4).

**TABLE 4:** Training and psychological strategies to facilitate negative splits.

Category	Key strategies
Progressive long runs	Start slow and finish fast to simulate race day effort
Tempo runs with fast finish	Maintain a steady pace and accelerate in the final minutes
Negative split intervals	Interval sessions with increasing pace across repetitions
Pacing awareness	Use HR monitors, GPS, or RPE to develop control and awareness
Mental skills training	Visualization, mindfulness, goal-setting, and positive self-talk
Race-day planning	Course familiarization, environmental adjustments, and pacing targets

## 5 Limitations and considerations in elite vs. recreational runners

While the physiological and psychological benefits of negative splits are well-documented, their practical application varies considerably between elite and recreational runners. Factors such as training status, experience, psychological resilience, and environmental conditions can all influence the feasibility and effectiveness of this pacing strategy.

Elite runners often possess superior aerobic capacity (VO_2max_), efficient running economy, and extensive race experience all of which facilitate precise pacing and energy distribution (Foster et al., 1994; Joyner and Coyle, 2008). Moreover, elite athletes typically race on flat, well-monitored courses with the support of pacemakers, time checks, and feedback tools, allowing for tight control over split times. These controlled conditions make negative splitting both more attainable and more advantageous at the top level of performance (Santos-Lozano et al., 2014; Hanley, 2015).

In contrast, recreational runners often lack the physiological conditioning and pacing awareness required to execute a disciplined start. They may begin races at an unsustainable effort due to overexcitement, crowd dynamics, or unrealistic time goals, resulting in rapid fatigue and a marked drop in pace the classic positive split pattern (Nikolaidis and Knechtle, 2017). Although wearable technologies such as GPS watches and heart rate monitors have become more affordable and widely adopted in recent years, effective usage still depends on runners’ experience and ability to interpret feedback (Lacey et al., 2022). A recent randomized controlled trial also showed that real-time feedback via wearable insoles significantly reduced injury risk, but performance improvements were contingent on active engagement with the technology (Van Hooren et al., 2024). Thus, while access to wearables is no longer a limiting factor for most non-elite runners, optimal pacing still requires structured training and pacing literacy.

Another important consideration is the course profile and environmental conditions. Hilly terrain, high temperatures, or crowded starting areas can disrupt pacing plans and make a strict negative split impractical or even counterproductive (González-Alonso et al., 1995; Ely et al., 2009). Runners must adapt pacing strategies to their individual context, experience level, and specific race goals.

Therefore, while the negative split offers a physiologically sound framework for performance optimization, it should not be viewed as universally optimal. Training specificity, psychological preparation, environmental planning, and realistic goal setting are essential prerequisites for its successful application across the full spectrum of marathon runners (Abbiss and Laursen, 2008; McCormick et al., 2015) (Table 5).

**TABLE 5:** Factors influencing negative split feasibility in elite vs. recreational runners.

Category	Elite runners	Recreational runners
Aerobic capacity and running economy	High VO_2max_ and efficient running economy	Lower VO_2max_, less efficient mechanics
Experience and discipline	Extensive racing experience and pacing discipline	Limited experience, prone to early overpacing
Race environment	Flat courses, pacemakers, feedback tools	Unpredictable conditions, limited real-time feedback
Training practices	Structured training with pacing strategies	Less structured, minimal pacing practice
Psychological readiness	Mental resilience, strategic patience	Overexcitement, goal misjudgment
External conditions	Usually optimized (weather, course)	Greater impact from heat, crowds, hills

## 6 Conclusion

Negative split pacing represents a physiologically supported and performance-enhancing strategy in marathon running. By distributing effort more conservatively in the early stages and accelerating in the latter half, runners can preserve muscle glycogen, maintain thermoregulatory and cardiovascular stability, and delay the onset of both central and peripheral fatigue. These advantages, coupled with reduced perceived exertion and enhanced psychological control, contribute to its success among elite marathoners and offer promising benefits for well-prepared recreational athletes.

Nonetheless, the effective implementation of a negative split strategy requires more than physiological capacity it demands targeted training interventions, pacing literacy, and mental discipline. Recreational runners, in particular, may benefit from progressive long runs, structured pacing sessions, and psychological techniques designed to promote controlled early efforts and confidence in finishing fast.

It is important to acknowledge, however, that this strategy is not universally applicable. Individual variability in fitness level, experience, race conditions, and terrain must be carefully considered when prescribing pacing approaches. For some runners, adaptive or even pacing strategies may be more suitable under specific contexts.

In conclusion, the negative split offers a compelling framework for optimizing endurance performance through a combination of physiological efficiency and strategic restraint. Future research and coaching practices should continue to explore its application across varying athlete populations, race profiles, and environmental demands to fully harness its potential for enhancing marathon outcomes.

## References

[B1] Abbiss C. R. Laursen P. B. (2008). Describing and understanding pacing strategies during athletic competition. Sports Med. 38, 239–252. 10.2165/00007256-200838030-00004 18278984

[B2] Barwood M. J. Thelwell R. C. Tipton M. J. (2008). Psychological skills training improves exercise performance in the heat. Med. Sci. Sports Exerc. 40, 387–396. 10.1249/mss.0b013e31815adf31 18202561

[B3] Bosquet L. Leger L. Legros P. (2002). Methods to determine aerobic endurance. Sports Med. 32, 675–700. 10.2165/00007256-200232110-00002 12196030

[B4] Brick N. MacIntyre T. Campbell M. (2015). Metacognitive processes in the self-regulation of performance in elite endurance runners. Psychol. Sport Exerc. 19, 1–9. 10.1016/j.psychsport.2015.02.003

[B5] Buman M. P. Omli J. W. Giacobbi P. R. Brewer B. W. (2008). Experiences and coping responses of “Hitting the Wall” for recreational marathon runners. J. Appl. Sport Psychol. 20, 282–300. 10.1080/10413200802078267

[B6] Coyle E. F. (1995). Substrate utilization during exercise in active people. Am. J. Clin. Nutr. 61, 968S-979S–979S. 10.1093/ajcn/61.4.968S 7900696

[B7] Coyle E. F. González-Alonso J. (2001). Cardiovascular drift during prolonged exercise: new perspectives. Exerc. Sport Sci. Rev. 29, 88–92. 10.1097/00003677-200104000-00009 11337829

[B8] Cuk I. Nikolaidis P. T. Villiger E. Knechtle B. (2021). Pacing in long-distance running: sex and age differences in 10-km race and marathon. Med. Kaunas. Lith. 57, 389. 10.3390/medicina57040389 PMC8073231 33920504

[B9] Darch L. Chalmers S. Wiltshire J. Causby R. Arnold J. (2022). Running-induced fatigue and impact loading in runners: a systematic review and meta-analysis. J. Sports Sci. 40, 1512–1531. 10.1080/02640414.2022.2089803 35723671

[B10] Del Coso J. Fernández D. Abián-Vicen J. Salinero J. J. González-Millán C. Areces F. (2013). Running pace decrease during a marathon is positively related to blood markers of muscle damage. PLoS One 8, e57602. 10.1371/journal.pone.0057602 23460881 PMC3583862

[B11] Ely B. R. Ely M. R. Cheuvront S. N. Kenefick R. W. DeGroot D. W. Montain S. J. (2009). Evidence against a 40 degrees C core temperature threshold for fatigue in humans. J. Appl. Physiol. 107, 1519–1525. 10.1152/japplphysiol.00577.2009 19713430

[B12] Esteve-Lanao J. Foster C. Seiler S. Lucia A. (2007). Impact of training intensity distribution on performance in endurance athletes. J. Strength Cond. Res. 21, 943–949. 10.1519/R-19725.1 17685689

[B13] Foster C. Schrager M. Snyder A. C. Thompson N. N. (1994). Pacing strategy and athletic performance. Sports Med. 17, 77–85. 10.2165/00007256-199417020-00001 8171225

[B14] González-Alonso J. Mora-Rodríguez R. Below P. R. Coyle E. F. (1995). Dehydration reduces cardiac output and increases systemic and cutaneous vascular resistance during exercise. J. Appl. Physiol. Bethesda Md 79, 1487–1496. 10.1152/jappl.1995.79.5.1487 8594004

[B15] González-Alonso J. Teller C. Andersen S. L. Jensen F. B. Hyldig T. Nielsen B. (1999). Influence of body temperature on the development of fatigue during prolonged exercise in the heat. J. Appl. Physiol. Bethesda Md 86, 1032–1039. 10.1152/jappl.1999.86.3.1032 10066720

[B16] Grivas G. V. Muniz-Pardos B. Guppy F. Pitsiladis A. Bundy R. Miller M. (2024). Assessing core body temperature in a cool marathon using two pill ingestion strategies. Transl. Exerc. Biomed. 1, 264–276. 10.1515/teb-2024-0012

[B17] Hanley B. (2015). Pacing profiles and pack running at the IAAF world half marathon championships. J. Sports Sci. 33, 1189–1195. 10.1080/02640414.2014.988742 25483017

[B18] Hargreaves M. Spriet L. L. (2020). Skeletal muscle energy metabolism during exercise. Nat. Metab. 2, 817–828. 10.1038/s42255-020-0251-4 32747792

[B19] Hazzaa W. A. Hottenrott L. Kamal M. A. Mattes K. (2023). The influence of general and local muscle fatigue on kinematics and plantar pressure distribution during running: a systematic review and meta-analysis. Sports 11, 241. 10.3390/sports11120241 38133108 PMC10747919

[B20] Joyner M. J. Coyle E. F. (2008). Endurance exercise performance: the physiology of champions. J. Physiol. 586, 35–44. 10.1113/jphysiol.2007.143834 17901124 PMC2375555

[B21] Joyner M. J. Ruiz J. R. Lucia A. (2011). The two-hour marathon: who and when? J. Appl. Physiol. Bethesda Md 110, 275–277. 10.1152/japplphysiol.00563.2010 20689089

[B22] Lacey A. Whyte E. O’Keeffe S. O’Connor S. Moran K. (2022). A qualitative examination of the factors affecting the adoption of injury focused wearable technologies in recreational runners. PloS One 17, e0265475. 10.1371/journal.pone.0265475 35793284 PMC9258862

[B23] McCormick A. Meijen C. Marcora S. (2015). Psychological determinants of whole-body endurance performance. Sports Med. Auckl. 45, 997–1015. 10.1007/s40279-015-0319-6 PMC4473096 25771784

[B24] Midgley A. W. McNaughton L. R. Wilkinson M. (2006). Is there an optimal training intensity for enhancing the maximal oxygen uptake of distance runners? empirical research findings, current opinions, physiological rationale and practical recommendations. Sports Med. Auckl. 36, 117–132. 10.2165/00007256-200636020-00003 16464121

[B25] Nikolaidis P. T. Knechtle B. (2017). Effect of age and performance on pacing of marathon runners. Open Access J. Sports Med. 8, 171–180. 10.2147/OAJSM.S141649 28860876 PMC5571841

[B26] Nikolaidis P. T. Knechtle B. (2018). Pacing strategies in the ‘athens classic marathon’: physiological and psychological aspects. Front. Physiol. 9, 1539. 10.3389/fphys.2018.01539 30450055 PMC6224376

[B27] Nikolaidis P. T. Knechtle B. (2024). Physiology of marathon: a narrative review of runners’ profile and predictors of performance. Physiologia 4, 317–326. 10.3390/physiologia4030019

[B28] Nybo L. (2008). Hyperthermia and fatigue. J. Appl. Physiol. Bethesda Md 104, 871–878. 10.1152/japplphysiol.00910.2007 17962572

[B29] Nybo L. Nielsen B. (2001). Hyperthermia and central fatigue during prolonged exercise in humans. J. Appl. Physiol. 91, 1055–1060. 10.1152/jappl.2001.91.3.1055 11509498

[B30] Nybo L. Rasmussen P. Sawka M. N. (2014). “Performance in the heat—physiological factors of importance for hyperthermia-induced fatigue,” in Comprehensive physiology (John Wiley and Sons, Ltd), 657–689. 10.1002/cphy.c130012 24715563

[B31] Oficial-Casado F. Uriel J. Jimenez-Perez I. Goethel M. F. Pérez-Soriano P. Priego-Quesada J. I. (2022). Consistency of pacing profile according to performance level in three different editions of the Chicago, London, and Tokyo marathons. Sci. Rep. 12, 10780. 10.1038/s41598-022-14868-6 35750788 PMC9232527

[B32] Prigent G. Apte S. Paraschiv-Ionescu A. Besson C. Gremeaux V. Aminian K. (2022). Concurrent evolution of biomechanical and physiological parameters with running-induced acute fatigue. Front. Physiol. 13, 814172. 10.3389/fphys.2022.814172 35222081 PMC8874325

[B33] Pycke J.-R. Billat V. (2022). Marathon performance depends on pacing oscillations between non symmetric extreme values. Int. J. Environ. Res. Public. Health 19, 2463. 10.3390/ijerph19042463 35206654 PMC8877899

[B34] Renfree A. St Clair Gibson A. (2013). Influence of different performance levels on pacing strategy during the Women’s world championship marathon race. Int. J. Sports Physiol. Perform. 8, 279–285. 10.1123/ijspp.8.3.279 23006811

[B35] Renfree A. West J. Corbett M. Rhoden C. St Clair Gibson A. (2012). Complex interplay between determinants of pacing and performance during 20-km cycle time trials. Int. J. Sports Physiol. Perform. 7, 121–129. 10.1123/ijspp.7.2.121 22173069

[B36] Ristanović L. Cuk I. Villiger E. Stojiljković S. Nikolaidis P. T. Weiss K. (2023). The pacing differences in performance levels of marathon and half-marathon runners. Front. Psychol. 14, 1273451. 10.3389/fpsyg.2023.1273451 38187410 PMC10771621

[B37] Santos-Lozano A. Collado P. S. Foster C. Lucia A. Garatachea N. (2014). Influence of sex and level on marathon pacing strategy. Insights from the New York city race. Int. J. Sports Med. 35, 933–938. 10.1055/s-0034-1367048 24886929

[B38] Sha J. Yi Q. Jiang X. Wang Z. Cao H. Jiang S. (2024). Pacing strategies in marathons: a systematic review. Heliyon 10, e36760. 10.1016/j.heliyon.2024.e36760 39281580 PMC11400961

[B39] Tucker R. Lambert M. I. Noakes T. D. (2006). An analysis of pacing strategies during men’s world-record performances in track athletics. Int. J. Sports Physiol. Perform. 1, 233–245. 10.1123/ijspp.1.3.233 19116437

[B40] Van Hooren B. Plasqui G. Meijer K. (2024). The effect of wearable-based real-time feedback on running injuries and running performance: a randomized controlled trial. Am. J. Sports Med. 52, 750–765. 10.1177/03635465231222464 38287728 PMC10905988

[B41] Zanini M. Folland J. P. Blagrove R. C. (2025). The effect of 90 and 120 min of running on the determinants of endurance performance in well‐trained Male marathon runners. Scand. J. Med. Sci. Sports 35, e70076. 10.1111/sms.70076 40375575 PMC12082016

